# Contrast sensitivity in Idiopathic Intracranial Hypertension

**DOI:** 10.22336/rjo.2020.59

**Published:** 2020

**Authors:** Obaidur Rehman, Parul Ichhpujani, Suresh Kumar

**Affiliations:** * Department of Ophthalmology, Government Medical College and Hospital, Chandigarh, India

**Keywords:** Idiopathic Intracranial Hypertension, contrast sensitivity, papilledema, visual function, SPARCS

## Abstract

**Background:** Idiopathic Intracranial Hypertension (IIH) is a disease of elevated intracranial pressure without any known cause. Visual dysfunction is the major morbidity of this disease but not much is known about the way the contrast sensitivity (CS) function is affected.

**Objective:** This prospective, interventional study attempted to evaluate the change in central and peripheral contrast sensitivity, after treatment in patients diagnosed with IIH.

**Materials and methods:** Twenty eyes of 10 IIH patients underwent an internet based, Spaeth Richman Contrast Sensitivity (SPARCS) test. Average and quadrant wise SPARCS scores were compared at presentation (treatment naïve), 1-month post treatment and 3 months post treatment.

**Results:** The average SPARCS scores pre-treatment, 1-month post-treatment and at 3 months post treatment were 68.8 + 10.16, 74.45 + 11.17 and 75.7 + 10.81 respectively. At 3 months visit, the average SPARCS score was nearly comparable to the average score in normal Indian subjects, observed in a previous study of ours. Quadrant wise change in contrast sensitivity from first visit to third visit was significant in superonasal (p=0.003), inferonasal (p=0.029) and inferotemporal (p= 0.007) quadrants.

**Discussion:** Effect of IIH on visual system is still a relatively unexplored area, especially in the Indians. Not many studies have concentrated on its impact on central as well as peripheral CS. Previous studies have hinted at a possible role of CS as a better indicator of visual dysfunction than other parameters.

**Conclusions:** IIH affects both central and peripheral contrast sensitivity and therapy results in the improvement of contrast deficit. Poor contrast can possibly be explained by relatively more involvement of Magnocellular pathway over the Parvocellular pathway.

**Abbreviations:** IIH = Idiopathic Intracranial Hypertension, CS = Contrast Sensitivity, SPARCS = Spaeth Richman Contrast Sensitivity Test, BMI = Body Mass Index, MC = Magnocellular pathway, PC = Parvocellular pathway

## Background

IIH is a neurological disorder, usually presenting with symptoms like headache, nausea, vomiting, tinnitus, but its major morbidity lies secondary to papilledema. Papilledema in IIH patients leads to optic nerve dysfunction and loss of sensory visual function, that can occur early or later in the course of this disease [**1**]. 

Testing solely Snellen visual acuity imparts only limited knowledge about visual function loss. Spatial vision loss may be assessed by testing visual contrast sensitivity (CS). CS is the visual ability to distinguish change in illumination between an object and its background. The possible role of CS, as an early indicator of compromised visual function, has been studied in glaucoma, cataract and various retinal disorders [**2**-**4**]. Loss of CS with preserved Snellen visual acuity has already been observed in diseases affecting afferent sensory visual function, such as multiple sclerosis, cerebral lesions and glaucoma [**5**-**7**]. Thus, assessment of visual acuity imparts knowledge of only one aspect of visual dysfunction and may not be a suitable indicator of subtle loss in visual function. By testing central and peripheral CS, visual dysfunction that is not explained by conventional techniques might also be explained. In a study by Wall and collaborators, out of various parameters tested in IIH, CS was the only visual parameter that correlated significantly with the symptom of sustained visual loss [**8**]. 

Most studies on visual function assessment in IIH have focused only on visual acuity and visual field testing, with only a few evaluating CS [**8**-**11**]. Thus, knowledge pertaining to the role of CS in IIH remains limited. None of the previous studies has provided follow up values and percentage changes with resolution of disc edema. Additionally, the studies evaluating contrast sensitivity have used conventional techniques of CS measurement, testing only central contrast. Our study evaluated central as well as peripheral CS in IIH patients, and noted the change seen with treatment, using an internet-based test relying on Weber’s contrast, the Spaeth Richman Contrast Sensitivity Test (SPARCS), as an evaluation tool. 

## Materials and methods

This pilot, prospective study examined 20 eyes of 10 consecutive treatment naïve IIH patients, who presented to the Ophthalmology or Neurology OPDs of the Government Medical College Hospital, Chandigarh, India. The study was in line with guidelines as set by the institutional ethics committee and an informed, written consent was obtained from all the patients prior to their enrolment. A detailed ophthalmological and neurological examination was carried out in all patients. The study conformed to the tenets of the Declaration of Helsinki.

All patients fulfilling the Modified Dandy’s criteria [**12**] were diagnosed to have IIH. These criteria included alert and awake patient, having signs of raised intracranial pressure, with no localizing sign, with normal neuroimaging (MRI) and having lumbar puncture opening pressures > 25 cm of water. If the CSF pressure was below 25 cm of water, the diagnosis of IIH was considered probable if all the other parameters were fulfilled. Imaging findings such as an empty sella, slit-like ventricles or “tight” subarachnoid spaces, supported the diagnosis.

All the patients aged above 18 years old, meeting the criteria and not having any concurrent ocular pathology, were enrolled in the study. 

A comprehensive ophthalmological examination was performed by a trained ophthalmologist and neurological examination was done by a trained neurologist. Ocular examination included visual acuity testing using a Snellen chart, detailed slit lamp examination including posterior segment examination with +90D lens. Grading of papilledema was done as per the Modified Frisen’s grading system [**13**]. 

Color vision testing was performed with pseudoisochromatic Ishihara’s chart (38 plates), perimetry using Humphrey visual ﬁeld analyzer (HVF 750i II Carl Zeiss Meditec Inc., Dublin, CA, USA), 24-2 SITA Fast protocol, contrast sensitivity with SPARCS, OCT Retinal Nerve Fibre Layer using optic nerve cube with Cirrus SD-OCT 500 machine (Carl Zeiss Inc.).

Body Mass Index (BMI) was calculated for each patient using the formula: weight (in kilograms)/ height (in meters)2.

Contrast enhanced MRI (Using Philips ACHIEVA, 1.5 Tesla) was done along with MR Venography and Post contrast T1, Post Contrast FLAIR and T2 Sag scans. A trained anaesthetist performed the diagnostic lumbar puncture in sterile operating room, after obtaining a written, informed consent. Patients were subsequently started on tablet Acetazolamide 250 mg thrice daily, post lumbar puncture. Follow up visits were planned at 1- and 3 months post the initiation of therapy. At each visit, visual acuity, central and peripheral contrast sensitivity, were evaluated. Oral Acetazolamide dose was tapered or adjuvant Topiramate (after neurologist consultation) was added as per the resolution of disc edema.

**Contrast sensitivity testing**

Contrast Sensitivity was tested separately for each eye in all the patients (with patients wearing their habitual glasses), using an internet-based SPARCS test, available at *https://www.sparcscontrastcenter.com*. SPARCS test was performed on a standard computer of 1024 x 768 resolution, 256 grey levels and a screen size of at least 22 cm wide and 26.5 cm height. 

The main investigator (OR) explained the test to patients in their vernacular language at the beginning of it. To avoid learning effects, two practice trials were conducted before the first baseline measurement and one before each subsequent visit. Test was always conducted in the same room with a LED light of 22 W, a Color Temperature of 6500 K, and Lumens of 1900 Lm. The room did not have any windows or daylight, to minimize glare.

The patient was seated 50 cm away from the screen, so that the test occupied 30° of horizontal vision and 23.5° of vertical vision. The patient was instructed to fixate on the central testing area, which subtended 5° horizontally and 3.5° vertically. The patient clicked on the central area when ready and had to identify vertical square wave gratings, which appeared randomly in any of the five testing areas (superonasal, superotemporal, inferonasal, inferotemporal or central). The gratings on the screen had a spatial frequency of 0.4 cycles per degree and lasted 0.3 seconds. The patient temporarily broke fixation to click on the area in which the grating was seen and subsequently fixed the central area again to get ready for the next grating, which appeared on the screen only after the patient clicked on the central testing area. The gratings appeared in a random manner and SPARCS recorded the correct or incorrect responses so as to establish the contrast threshold.

The contrast value was calculated by Weber contrast. The central area and four peripheral areas each received separate scores, 100 being the perfect total score (score of 20 in each quadrant). Each log-based score was then scaled out of 20 by dividing by 2.35 and multiplying by 20. The total SPARCS score was calculated by addition of score from each individual testing area.

**Statistical Analysis**

Statistical analysis was done using IBM SPSS version 22 (IBM Corp, 2013. SPSS for Windows. Armonk, NY: IBM Corp.). 

Descriptive analysis involved mean and standard deviation for all quantitative variables, and proportion and frequency for all categorical variables. All quantitative variables were assessed for normal distribution within each category of explanatory variable. Shapiro Wilk test was also performed to check normal distribution and a P value of > 0.05 was accepted as normal distribution.

Association between quantitative explanatory and outcome variables was assessed by calculating Pearson correlation coefficient and the data was represented in a scatter diagram. P value < 0.05 was accepted as statistically significant.

## Results

**Basic Demographics:** Data of 20 eyes (10 patients: 2 males; 8 females) were included in our study. The average age of presentation was 36.1 ± 12.93 years (range 21-62 years). However, if we looked only at the female patients, the average age of presentation was 23.4 + 10.07 years (21-46 years). 

**Fig. 1:**
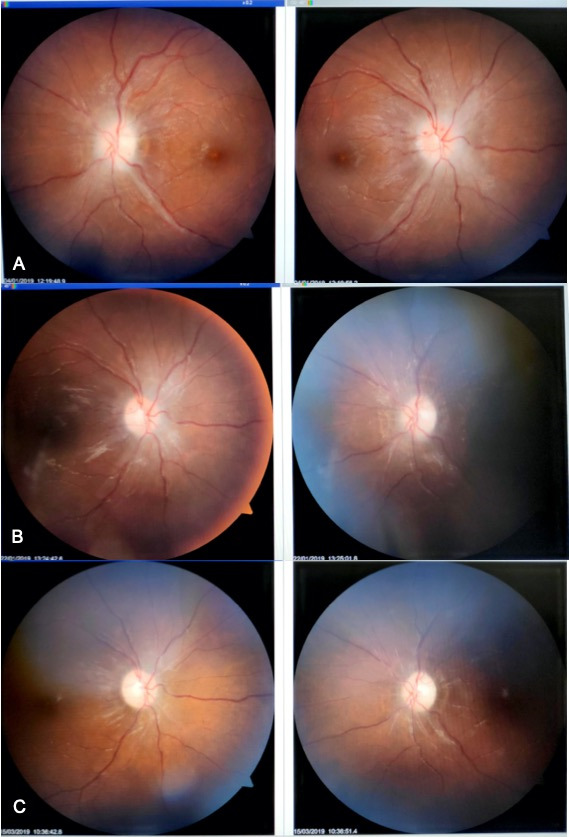
A representative case showing resolution of papilledema. A: First visit; B: Second visit; C: Third visit

**Presentation:** Headache and blurred vision were reported by all patients (100%), dizziness and nausea by 5 (50%), back and neck pain by 3 (30%) and tinnitus by 2 (20%) patients. Symmetrical papilledema in both eyes was noted in all the patients. At the end of the 3 months follow-up period, 11 eyes still showed presence of grade 1 papilledema while 9 eyes had complete resolution of papilledema. Higher grade of papilledema (grade 4 and 5) was observed in 8 eyes and they showed a faster response to treatment (**Fig. 1**).

**BMI:** The average Body Mass Index was 25.09 kg/ m2 (range 21.87-33.2). However, if only the female gender was considered, the average BMI was 28.70 kg/ m2 (range 23.5-33.2). Both of these values fall into the pre-obese category (range of BMI = 25-29.9) as per WHO criteria for Asian population.

**Systemic History:** Hypertension had been recently diagnosed in 1 patient, while 1 patient had hypothyroidism for past 2 years and 1 patient was an old case of deep vein thrombosis. No known systemic illness was present in any other patient.

**Medication History:** Intake of analgesics was reported by 2 patients (Tab Paracetamol 500 mg SOS), 1 patient reported intake of anti-hypertensive drug (Tab Amlodipine 5 mg OD), 1 patient had history of intake combined oral contraceptive pills 1 month back, 1 patient was taking anti-epileptic drug (Tab Topiramate 25 mg BD) and 1 patient was taking thyroid medication (Tab Thyroxine 50 µm OD). 

**Visual acuity:** Best corrected visual acuity (Snellen chart) was noted to be 6/ 6 in 65% of the eyes (n = 13), 6/ 9 in 15% (n = 3), 6/ 12 in 10% (n=2), 6/ 18 in 5% (n=1) and 6/ 36 in 5% of the eyes (n=1) at presentation. 

**Contrast sensitivity:** As for a normative database of SPARCS scores, it is yet to be established for the Indian population, and we used data from a previous study done at our centre by Thakur and coworkers [**14**], who had evaluated SPARCS in glaucoma patients and normal subjects. In that study, the average total SPARCS score was found to be 76.02 + 6.35 (range 61.0-86.5) amongst 45 controls (normals) in Indian population. In the present study, the mean percentage increase in average SPARCS score was noted to be 8.84% from first to second visit and 10.73% from first to third visit. Scores of individual quadrants in SPARCS for each visit are presented in **Table 1**. Total SPARCS scores in both eyes of a representative case at each visit are presented in **Table 2**.

**Table 1:** Scores of individual quadrants in SPARCS for each visit

Quadrant	Normal Score (Thakur et al.)	First Visit Score	Second visit Score	Third visit Score	% change (First to Third)
Superotemporal	15.96 + 1.97	15.47 + 3.12	15.96 + 3.04	15.68 + 2.08	4.93
Superonasal	15.98 + 1.67	14.78 + 2.57	15.48 + 2.14	16.29 + 2.34	12.04
Central	15.15 + 2.24	13.66 + 2.55	14.55 + 3.53	14.14 + 2.71	6.95
Inferotemporal	14.42 + 1.74	12.92 + 2.24	13.93 + 1.79	14.77 + 2.87	16.2
Inferonasal	14.45 + 2.12	12.95 + 2.32	14.61 + 2.65	14.57 + 2.62	15.93
Total	76.02 + 6.35	68.8 + 10.16	74.45 + 11.17	75.7 + 10.81	10.73

Analysis of individual values revealed quadrant wise change from first visit to third visit was significant in superonasal (p=0.003), inferonasal (p=0.029) and inferotemporal (p=0.007) quadrants. It was noted to be non-significant in superotemporal (p= 0.36) and central (p= 0.116) quadrants.

**Table 2:** Total SPARCS scores of a representative case at each visit

	First Visit Score	Second visit Score	Third visit Score	% change (First to Third)
Right eye	76	77	83	9.2
Left eye	53	71	76	43.3

Significant change (p<0.05) was noted in total SPARCS scores with resolution of papilledema

## Discussion

Idiopathic Intracranial Hypertension has a reported worldwide incidence of 0.9/ 100,000 population and it further rises to 3.5/ 100,000 when female population 15-44 years of age is taken into account [**15**]. Currently, there are no studies on Indian population, so the incidence remains unknown in India. Bruce BB et al. studied IIH in 450 patients and found the prevalence of IIH in men to be 10% [**16**]. Although our study had a small sample, it also had a female majority (8 females; 80%).

Wall and coworkers were the first to evaluate CS in IIH evaluating 12 patients using six Arden grating plates [**9**]. CS loss was detected in 9/ 12 patients (75%) and 13/ 24 eyes (54%). It was noted that CS improved as papilledema resolved. The authors concluded that CS was useful for visual loss detection and for serial follow up of patients with IIH. However, the chief drawback of their study was that only 9/ 12 patients had active disease at the time of testing while other 3 had resolved papilledema. Additionally, Arden plates are not a forced choice method, hence results vary as exposure speed of plate is varied by the examiner.

Bulens tested 20 patients of IIH using sine wave gratings on a screen, and, after comparison with a control population, found CS loss in 43% of the patients. They noted visual impairment attributable to IIH in 16% of the patients, thereby concluding that visual loss may occur even in preserved Snellen acuity and CS was a useful tool [**10**]. The CS test technique had similar drawbacks as that of Arden plates.

Later, Wall and George prospectively tested various visual parameters in 50 IIH patients. CS was measured using Arden plates initially and then Vistech Contrast test chart in the last 4 years of the study. CS abnormality was seen in 50% of the patients and Snellen acuity in 22%, implying that CS was more sensitive in picking up changes than Snellen visual acuity [**8**]. This study lacked uniformity in testing and follow up as two different tests were used in different time periods. Additionally, Vistech test is not a forced choice test and has low reliability. 

Rowe and Sarkies studied 35 patients of IIH (from 1993 to 1996) and followed up these patients for 3 years. The Pelli Robson chart was employed for CS testing and they could find no significant difference between CS and visual acuity while determination of visual deficit.

They also commented that a better detailed computerized assessment of contrast sensitivity may prove to be more sensitive for visual deficit detection [**11**]. 

None of the previously reported studies was either computerized or considered peripheral CS.

In order for better comprehension of the reason for decline in CS in IIH, a revisit to neuroanatomy is needed. Three major neural retinogeniculate pathways have been identified in modern studies; they rely on information from the retina to the visual cortex – the magnocellular pathway (MC), the parvocellular pathway (PC) and the koniocellular (KC) pathway [**17**,**18**]. Each of these has distinctive characteristics and is related to different aspects of vision. The MC pathway has high temporal frequency sensitivity, showing much more sensitivity to low spatial, high temporal frequencies. It is considered to be responsible for the detection of contrast over a wide range of luminances. The PC pathway has a greater sensitivity for high spatial, low temporal frequencies and is mainly involved in chromatic processing and visual acuity.

We suggested that the engorgement of retinal ganglion cells at the optic nerve head, relying on the MC and PC pathways, due to disc edema and their subsequent dysfunction, may result in contrast sensitivity loss. Additionally, improvement in total and average SPARCS scores, with resolving papilledema, points towards the same. A higher cause, such as a cerebral cause, seems unlikely as no intracranial disturbances were observed in any of the patients. 

Additionally, as most of the patients had a good best corrected visual acuity, with 65% eyes having 6/ 6 Snellen acuity, it appears that it is the MC pathway that is more affected than the PC pathway. 

All patients reported improvement in vision related symptoms, underlining the importance of CS as a measure of ocular morbidity. At the end of three month follow up, the average total SPARCS score was nearly the same as that of healthy subjects in Thakur’s study, confirming the effectiveness of therapy and resolution of contrast deficit. 

55% eyes still had papilledema grade 1 at the 3 months follow up after therapy initiation. All patients are still under follow up and a timeline for resolution of papilledema and possible factors influencing it still need to be studied. 

## Conclusions

IIH impacts central as well as peripheral CS. Improvement in average total SPARCS score and in the average score of each quadrant, with therapy and subsequent resolution of edema, was seen. Changes in CS are often unnoticed by the patients and undocumented by the physician. It is therefore important to continue monitoring CS throughout the routine follow-up for the detection of visual function loss and any subtle change on subsequent visits.

**Conflict of interest**

Nil.

**Informed Consent**

Informed consent has been obtained from all individuals included in this study.

**Authorization for the use of human subjects**

The research related to human use complies with all the relevant national regulations, institutional policies, is in accordance with the tenets of the Helsinki Declaration, and has been approved by the Ethics Committee of the Government Medical College Hospital, Chandigarh, India.

**Acknowledgements**

Nil.

**Sources of funding**

Nil.

**Disclosures**

Nil.

**Presentation at a meeting**

Nil.
